# Fido, a Novel AMPylation Domain Common to Fic, Doc, and AvrB

**DOI:** 10.1371/journal.pone.0005818

**Published:** 2009-06-05

**Authors:** Lisa N. Kinch, Melanie L. Yarbrough, Kim Orth, Nick V. Grishin

**Affiliations:** 1 Howard Hughes Medical Institute, University of Texas Southwestern Medical Center, Dallas, Texas, United States of America; 2 Department of Biochemistry, University of Texas Southwestern Medical Center, Dallas, Texas, United States of America; 3 Department of Molecular Biology, University of Texas Southwestern Medical Center, Dallas, Texas, United States of America; University of Queensland, Australia

## Abstract

**Background:**

The *Vibrio parahaemolyticus* type III secreted effector VopS contains a fic domain that covalently modifies Rho GTPase threonine with AMP to inhibit downstream signaling events in host cells. The VopS fic domain includes a conserved sequence motif (HPFx[D/E]GN[G/K]R) that contributes to AMPylation. Fic domains are found in a variety of species, including bacteria, a few archaea, and metazoan eukaryotes.

**Methodology/Principal Findings:**

We show that the AMPylation activity extends to a eukaryotic fic domain in *Drosophila melanogaster* CG9523, and use sequence and structure based computational methods to identify related domains in doc toxins and the type III effector AvrB. The conserved sequence motif that contributes to AMPylation unites fic with doc. Although AvrB lacks this motif, its structure reveals a similar topology to the fic and doc folds. AvrB binds to a peptide fragment of its host virulence target in a similar manner as fic binds peptide substrate. AvrB also orients a phosphate group from a bound ADP ligand near the peptide-binding site and in a similar position as a bound fic phosphate.

**Conclusions/Significance:**

The demonstrated eukaryotic fic domain AMPylation activity suggests that the VopS effector has exploited a novel host posttranslational modification. Fic domain-related structures give insight to the AMPylation active site and to the VopS fic domain interaction with its host GTPase target. These results suggest that fic, doc, and AvrB stem from a common ancestor that has evolved to AMPylate protein substrates.

## Introduction

The molecular function of fic (filamentation induced by cAMP) domains has remained elusive until recently. The original description of fic derives from the *E. coli fic-1* gene required for cAMP-induced filamentation [1], [2]. Bacterial effector proteins that serve to modulate the activity of host cells also include fic domains. For example, the type IV secretion system effector AnkX from *L. pneumophila* has an N-terminal fic domain required for disrupting host secretory vesicle transport [3]. A fic domain is also present in VopS, a type III secretion system effector from *V. parahaemolyticus* that causes eukaryotic cell cytotoxicity [4]. The fic domain of VopS was shown to covalently modify host Rho GTPases with AMP. This AMPylation reaction occurred on a conserved threonine residue located in the GTPase Switch I region responsible for binding downstream effectors. Mutation of a conserved histidine in VopS abrogated this function, suggesting an enzymatic role for the residue in AMPylation [5].

AMPylation represents a newly discovered posttranslational modification used to stably modify proteins with AMP. This signaling mechanism is predicted to be functionally similar to other posttranslation modifications such as phosphorylation, SUMOylation or acetylation, because the added moiety changes the activity of the modified protein. The covalent attachment of AMP by a phosphodiester bond is predicted to be reversible and is bulky enough to provide a docking site for a putative AMP binding domain. Although a fic domain-containing protein is known to catalyze the AMPylation reaction, other components involved in this signaling system are yet to be discovered.

The fic domain is classified together with a second family of sequences, doc (death on curing), in the protein families database PFAM [6], and the sequences have been linked previously [7]. The combined family contains a central motif (fido) conserved in most sequences (HPFx[D/E]GN[G/K]R), with the motif His contributing to fic AMPylation [5]. Doc belongs to a toxin-antitoxin module found on *E.coli* phage P1 that acts as a plasmid addiction system. In the absence of phd antitoxin, doc induces growth arrest of *E.coli* by targeting protein translation elongation [8], [9]. Mutation of the motif His renders doc nontoxic, suggesting that the motif represents a functional site common to both families [10].

Structural genomics efforts have provided coordinates for four different hypothetical proteins with fic domains (2f6s, 2g03, 3cuc, and 3eqx), revealing a common α-helical fold topology [11]–[14]. Two of the fic domains (2f6s and 2g03) are related to the others by a circularly permuted helix. A similar α-helical structure of a non-toxic doc mutant (H66Y) bound to phd antitoxin (3dd7) replaces the permuted fic α-helix with an α-helical segment of phd. Together with the functional motif, the common topology of fic and doc domains suggests their evolution from a common ancestor [10].

To help resolve fido classification, we explored evolutionary relationships using a combination of sequence-based and structure-based methods. Sequence evidence supports an evolutionary link between fic and doc, while structural evidence supports a homologous relationship between these two families and a domain from the *Pseudomonas syringae* type III effector protein avirulence protein B (AvrB), which is delivered into plant cells to illicit accelerated defense responses and hypersensitive cell death by targeting the host immune resistance protein RIN4. We propose to unite domains from these three families (Fic, Doc, and AvrB) into a single superfamily (fido) that likely perform similar signaling functions. We show that like the VopS fic domain, a *Drosophila melanogaster* fic homolog self-AMPylates.

## Results and Discussion

### Classification of fido structures suggests homology to AvrB

**Figure 1:**
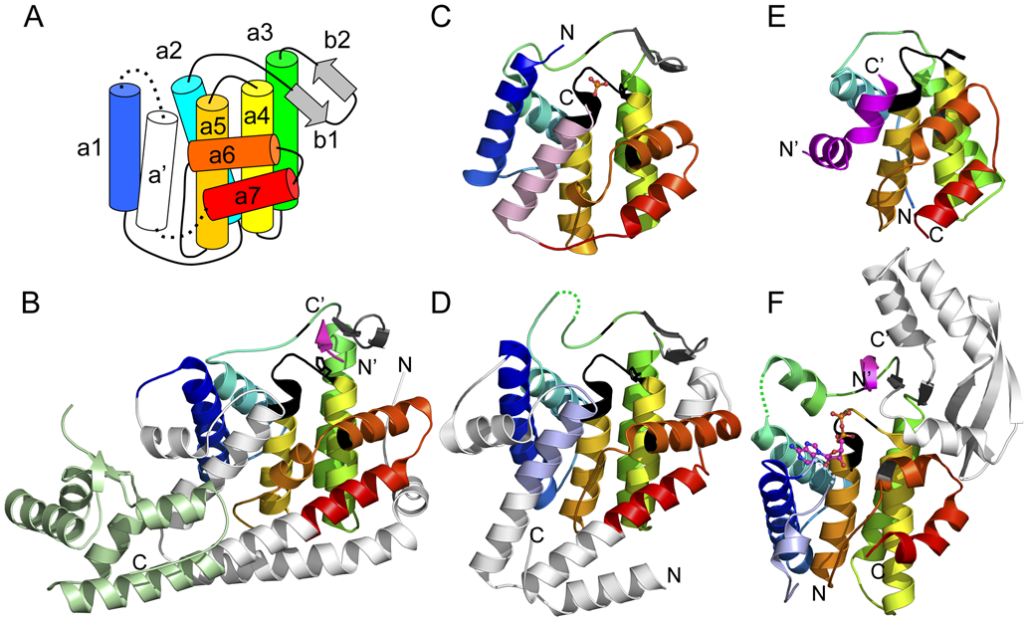
Structural similarities of fido domain-containing families. Structural models of fic homologs define a core fic domain secondary structure topology labeled from N-terminus to C-terminus (A). Diverse fic domain-containing structures are illustrated from a *Shewanella oneidensis* [2qc0] (B), *Helicobacter pylori* [2f6s] (C), and *Bacteroides thetaiotaomicron* [3cuc] (D). The common core α-helices are colored in rainbow from N-terminus (blue) to C-terminus (red). A permuted helix is colored pink (contributed from the C-terminus) or slate (contributed from the N-terminus). Extended elements that decorate the core are colored white, a helix-turn-helix domain is colored light green, and a β-hairpin that binds peptide ligand (magenta) is colored gray. Bound ligands are represented as ball-and-stick and conserved sequence motifs marking the active sites are black. The N-terminus and the C-terminus of each structure is labeled. Similar structures retain most or all of the fic domain core: a doc structure [3dd7] bound to phd antitoxin (magenta) (E) and an AvrB structure bound to Rin4 peptide (magenta) [2nud] also binds ADP (ball-and-stick) [2nun, superimposed] (F).

Comparison of four available fic domain-containing hypothetical protein structures reveals a common fold topology that contributes to the evolutionary core of the domain (Figure 1A). The fic domain core includes eight α-helices, arranged as a six-helix up and down bundle (a1 - a5 and a') decorated by two additional helices (a6 and a7) lying almost perpendicular to the bundle. A β-hairpin inserted between helix a2 and a3 is positioned near the fido motif (loop between a4 and a5). In one fic-containing structure from *Shewanella oneidensis* (Figure 1B), a peptide from the N-terminus of a neighboring chain forms a β-like interaction with the hairpin [14]. The fic core of this structure is decorated by additional helices and includes a C-terminal domain not found in the other structures. One of the core α-helices (a') is permuted when compared to two closely related fic domains from *Helicobacter pylori* (Figure 1C) and *Neisseria meningitidis* (not shown), which contribute the a' helix from the C-terminus. Both the *Shewanella oneidensis* fic and the *Bacteroides thetaiotaomicron* fic (Figure 1D) include permuted a' helices contributed from the N-terminus and similar decorating helices.

A doc domain structure bound to phd antitoxin revealed a similar overall topology to the fic domain [10]. However, doc lacks a few elements of the conserved fic core, including the first and the permuted α-helices and the β-hairpin (Figure 1E). The phd antitoxin replaces the two missing core α-helices, and was suggested to complete the core through fold complementation. Given the similar overall fold and the common motif positioned between corresponding α-helices, doc was suggested to have evolved from fic [10].

The fido structures display a similar topology that is somewhat unique among existing folds. To determine the degree of structural similarity between these folds and to help resolve existing classification schemes, we chose to compare each structure to existing structures in the PDB. The top hits (ordered by Dali Z-score) for each fic domain-containing query correspond to representative fic structures (Table 1). Despite recognized similarity to doc, the next best hit in each case represents a type III effector protein structure AvrB from Pseudomonas syringae. The AvrB structure is also the top hit using doc as a query, followed by the fic domains. Thus the fic, doc and AvrB structures form a “closed structural group”, displaying closer similarity to each other than to any other known protein structure. This property of structural similarity has been used previously to delineate EDD and SHS2 domains [15], [16] and is suggestive of monophyly.

**Table 1:** Structural Similarity Search**.

*Query*	*Hit*		*DaliLite*
PDB	PDB	family	*Z-score*
2f6s	2f6s	fic	37.6
	2g03	fic	32.6
	3cuc	fic	12.2
	2qc0	fic	12
	2nud	AvrB	8.1
	3dd7	doc	4
2g03	2g03	fic	37.3
	2f6s	fic	32.6
	2qc0	fic	12.2
	3cuc	fic	12
	2nud	AvrB	8.3
	3dd7	doc	6.9
2qc0	2qc0	fic	61.8
	3cuc	fic	20.1
	2g03	fic	12.2
	2f6s	fic	12.1
	2nud	AvrB	10.6
	3dd7*	doc	6.8
3cuc	3cuc	fic	47
	2qc0	fic	20.1
	2f6s	fic	12.2
	2g03	fic	12
	2nud	AvrB	11.3
	3dd7	doc	7.8
3dd7	3dd7	doc	29
	2nud	AvrB	8.3
	3cuc	fic	7.8
	2f6s	fic	7.6
	2g03	fic	7.5
	2qc0	fic	6.9
2nud	2nud	AvrB	49.8
	3cuc	fic	11.3
	2qc0	fic	10.5
	2f6s	fic	8.3
	3dd7	doc	8.1
	2g03	fic	8.1

* Structure hit to fido domain follows hits to C-terminal domain with HTH (ie. 2fe3, Z-score 7.3).

While the apo AvrB structure represented a novel fold with no recognizable homologs upon its release [17], a subsequent publication of RIN4 peptide-bound and nucleotide-bound AvrB structures [18] failed to consider the presence of fic domain structures in the PDB. AvrB retains all elements of the fic domain core, including the α-helices and β-hairpin missing in doc (Figure 1F). An additional domain is inserted in the β-hairpin that extends it into a five-stranded sheet referred to as the upper lobe [17]. A structure of AvrB bound to Rin4 reveals a similar peptide-binding mode as found in one of the fic structures (Figure 1B). The Rin4 peptide forms a β-like interaction with the extended sheet. Although the AvrB sequence lacks a number of the conserved fido-motif residues, it retains the small Pro and Gly motif residues that allow AvrB to adopt a similar loop conformation as in fic and doc structures (Figure 1, black). A number of AvrB mutations have been shown to alter the binding of Rin4. Two mutations in the upper lobe β-sheet (T125A and Q164H) and one mutation in the active site loop (S268I) abolish Rin4 binding, while another mutation in the active site pocket (D297A) enhances binding 10-fold [19]. Interestingly, the catalytically inactive VopS mutation also enhances its binding to GTPase by a “substrate trap” [5].

### Sequence-based support for fido classification

To help confirm the implied evolutionary link between fic and doc, we used sequences corresponding to each domain as queries in transitive searches with PSI-BLAST [20]. The representative fic domain sequence from the *B. thetaiotaomicron* structure identified a number of doc sequences (ie. gi|159046642, detected in iteration 4, E-value 0.002) and converged without identifying sequences from other families. Similarly, the representative doc domain structure sequence identified a number of fic sequences (ie. gi| 195116213, detected in iteration 3, E-value 2e^−04^). The PSI-BLAST hits encompass a significant portion of the fic core and identified the conserved fido sequence motif (HPFx[D/E]GN[G/K]R). Identified fic and doc domain-containing sequences are present in genomes from bacteria, archaea, and eukaryota, with the latter distributed among metazoa species (HypE in human) and a few fungi. The phylogenetic distribution suggests extensive horizontal gene transfer (Figure S1), which represents a common observance for virulence and other genes involved in fitness [21].

Despite the structure similarity between AvrB and fido, most sequence methods fail to identify connecting links. However, using AvrB as a sequence query with the sensitive profile-based homology detection method COMPASS [22], [23] identified an insignificant hit (E-value 5) to the fic domain structure (3cuc). COMPASS accurately aligns the sequences of the two structures C-terminal to the inserted AvrB upper lobe domain, including the second strand of the β-hairpin, the active site loop, and helices a3 - a5. Apparently, the presence of an inserted domain within the AvrB β-hairpin, combined with an alteration of several active site motif residues hinders sequence recognition. Submitting an AvrB sequence that lacks the inserted domain to a fold-recognition meta-server (http://meta.bioinfo.pl) identifies fic domain structures as top hits with 3D-Jury scores [24] ranging from 48.83 for 3cuc to 60.83 for 2qc0 (scores correspond to approximately 50–60 correctly predicted residues [25]). The individual servers that identified fic domains from the truncated AvrB sequence (BASIC [26], SamT02 [27], and FFAS03 [28]) contain significant homology detection components.

**Figure 2:**
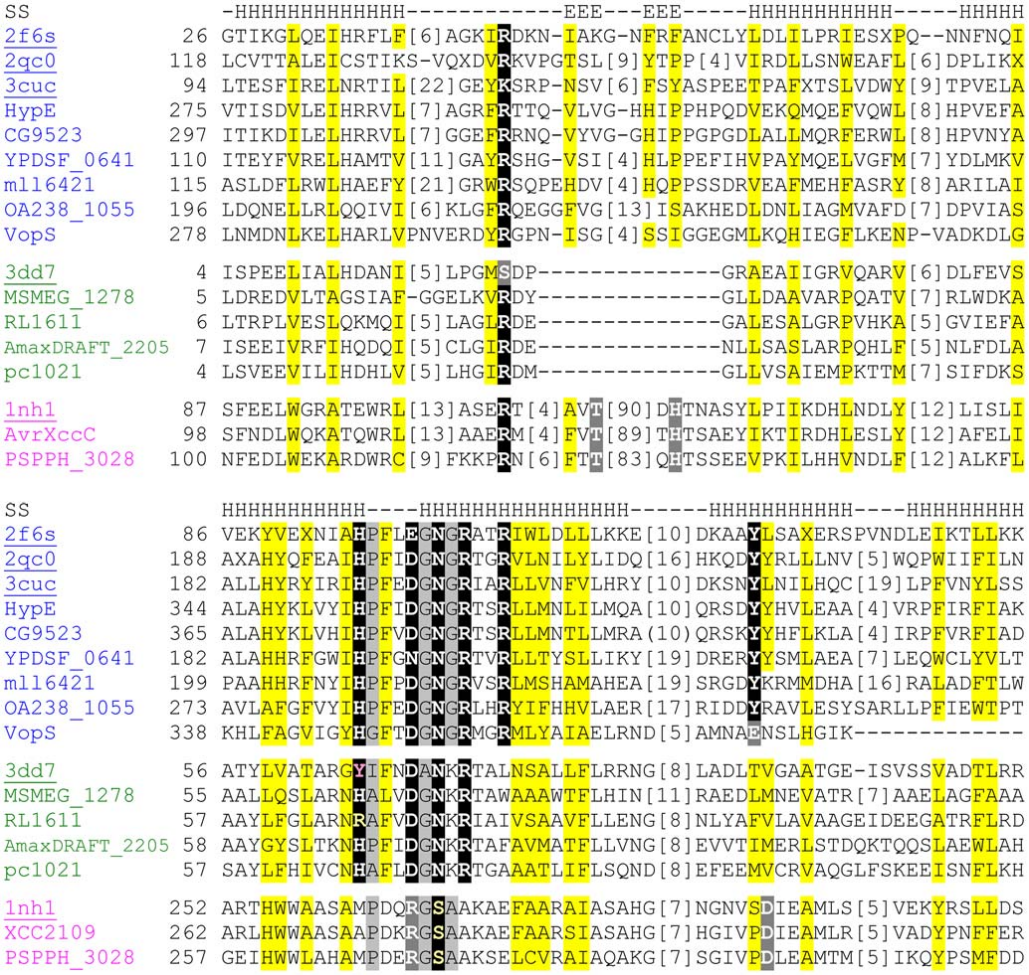
Multiple sequence alignment of fido structures with representative sequences. Fic, doc, and AvrB sequences are colored (blue, green, and magenta, respectively) and labeled with PDB ID (underlined), locus tag, or protein name. Positions corresponding to structurally conserved secondary structural elements are marked above the alignment (E for β-strand and H for α-helix). The number of the first residue position is indicated before each sequence, while omitted residues are in brackets. Uncharged residues at mainly hydrophobic positions are highlighted in yellow, and conserved small residues are highlighted in light gray. Conserved fic polar residues are highlighted black, along with the conservations that extended to doc or AvrB sequences. Moderate changes at conserved fic positions and conserved polar positions in AvrB that may represent active site migrations are highlighted in dark gray.

The multiple sequence alignment illustrated in Figure 2 highlights the conservation patterns of representative fido domains. Each family displays similar hydrophobicity patterns (yellow highlights) corresponding to the secondary structural elements of the core fold. Conserved polar residues (black highlights) can be mapped to the structures: an R follows the α-helix a2, the fido motif HisPFx[D/E]G forms a loop connecting the two central α-helices a3 and a4, and the fido motif N[G/K]R forms the N-terminus of α-helix a4. A number of conserved positions in fic display different conservations in AvrB. The fido [D/E] is a conserved R in AvrB, while the fido N is a conserved S. A conserved Y in the α-helix a5 is positioned near a D in AvrB. In multiple sequence alignments of distant homologs, a similar “conservation of conservations” often reflects common functional sites [29]. Accordingly, mutation of AvrB R266 (corresponding to fido D/E) and D297 cause a loss of function leading to an abrogation of hypersensitive response in host plant cells, while mutation of T125 and H217 located in the peptide-binding β-hairpin abrogate both Rin4 interaction and AvrB induced function [18], [19].

### Fido domain organization suggests roles in stress response and cholesterol metabolism

**Figure 3:**
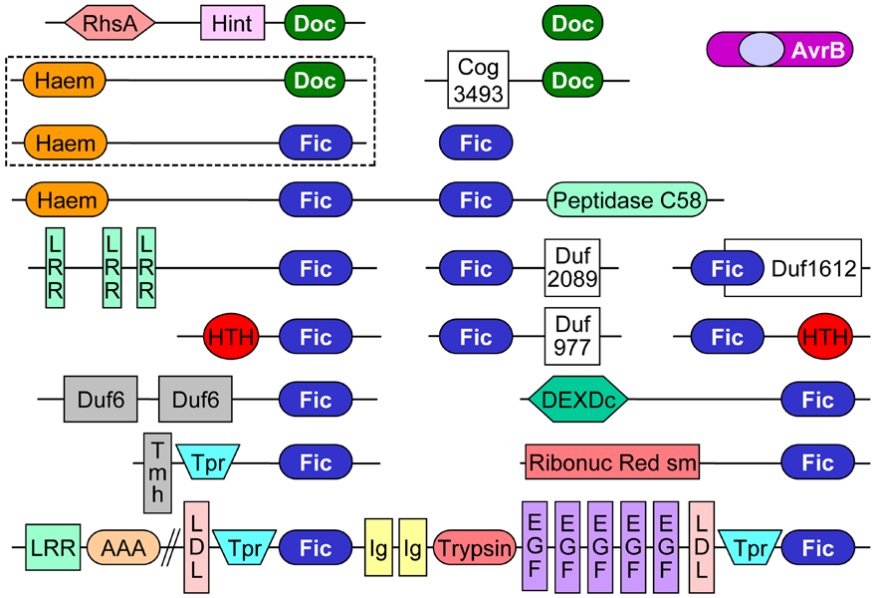
Fido Domains. The domain organization of representative doc (green rectangle), fic (blue rectangle), and AvrB (magenta rounded rectangle) sequences is depicted. Abbreviations: RhsA, rhs repeat family protein; Hint, hedgehog/intein domain; Haem, haemagglutination activity domain; LRR, leucine rich repeat; HTH, regulatory helix-turn-helix; Tmh, transmembrane helix; Tpr, tetratrico peptide repeat; AAA, ATPase containing von Willebrand factor type A domain; LDL, Low-density lipoprotein receptor domain class A; Ig, Immunoglobulin-like; Trypsin, trypsin-like serine protease; EGF, calcium binding epidermal growth factor-like domain; Ribonuc Red sm, ribonucleotide reductase small subunit; DEXDc, DEAD-like helicase.

Similar to other signaling proteins, fic and doc are fused to a number of different domains that might function in AMPlyation pathways (Figure 3). The *Haemophilus somnus* virulence factor p76 (ibpA) includes an N-terminal haemagglutination activity domain (Haem), two fic domains, and a papain-like protease (peptidase C58) also found in YopT and AvrPphB avirulence proteins. Like VopS, YopT disrupts the actin cytoskeleton of the host cell by inhibiting Rho GTPase signaling. YopT achieves this inhibition by cleaving Rho GTPases after a post-translationally modified cysteine, which releases the GTPases from the membrane where they function [30]. Thus, the *H. somnus* virulence factor includes multiple domains that modify the same signaling pathway. This functional domain organization suggests that other fusions might give similar clues to activity or help identify additional signaling components of AMPylation. The ibpA Haem domain is also found at the N-terminus of a doc domain containing protein from *Comamonas testosteroni*, providing further support for the homology and functional similarity between fic and doc.

A number of fusions exist between fic domains and helix-turn-helix (HTH) DNA-binding domains. These HTH domains may regulate expression of fic or other components of AMPylation signaling. The DNA-binding component of these HTH domains is reminiscent of the phd antitoxin N-terminal DNA-binding domain that serves to autorepresses the doc toxin-antitoxin operon [31]. A majority of the HTH domains belong to the arsenical resistance operon repressor subfamily, indicating a potential role of fic in stress responses. We identify several additional HTH folds among domains of unknown function fused to fic (duf977, duf1612, and duf2089 in Figure 3). HHpred and COMPASS searches identify HTH folds as top significant hits to these domains: duf977 and the C-terminus of duf1612 find “winged helix” DNA-binding domains such as the 1j5y N-terminus (COMPASS E-value 5.84e-18) and the 1z05 N-terminus (COMPASS E-value 1.89e-14), while duf2089 finds C-terminal effector domains of the bipartite response regulators such as 1je8 (COMPASS E-value 6.97e-04). Interestingly, the N-terminus of duf1612 corresponds to remote fic-like sequences, and a member of this duf, *Rhizobium* Y4CF, belongs to a replicon that allows bacteria to interact symbiotically with leguminous plants [32]. The C-terminal domain from the *S. oneidensis* fic structure (Figure 2B, light green) represents a winged helix-turn-helix that is not recognized by sequence.

Eukaryotic fic sequences such as HypE contain a predicted N-terminal transmembrane helix followed by Tpr repeats. One interesting eukaryotic sequence from *Branchiostoma floridae* includes several additional domains that might hint at components involved in the eukaryotic fic domain signaling process. In addition to two fic and two Tpr, the *B. floridae* protein domain organization includes two low density lipoprotein receptor class a (LDL), two immunoglobulin (Ig), a trypsin-like serine protease (Trypsin), and five repeated calcium-binding EGF-like domains (EGF). The presence of these domains indicates an extracellular or intra vesicular localization of the pathway in eukaryotes, and the LDL might suggest involvment of AMPylation in cholesterol metabolism or trafficking.

### Functional implications of Fido classification

**Figure 4:**
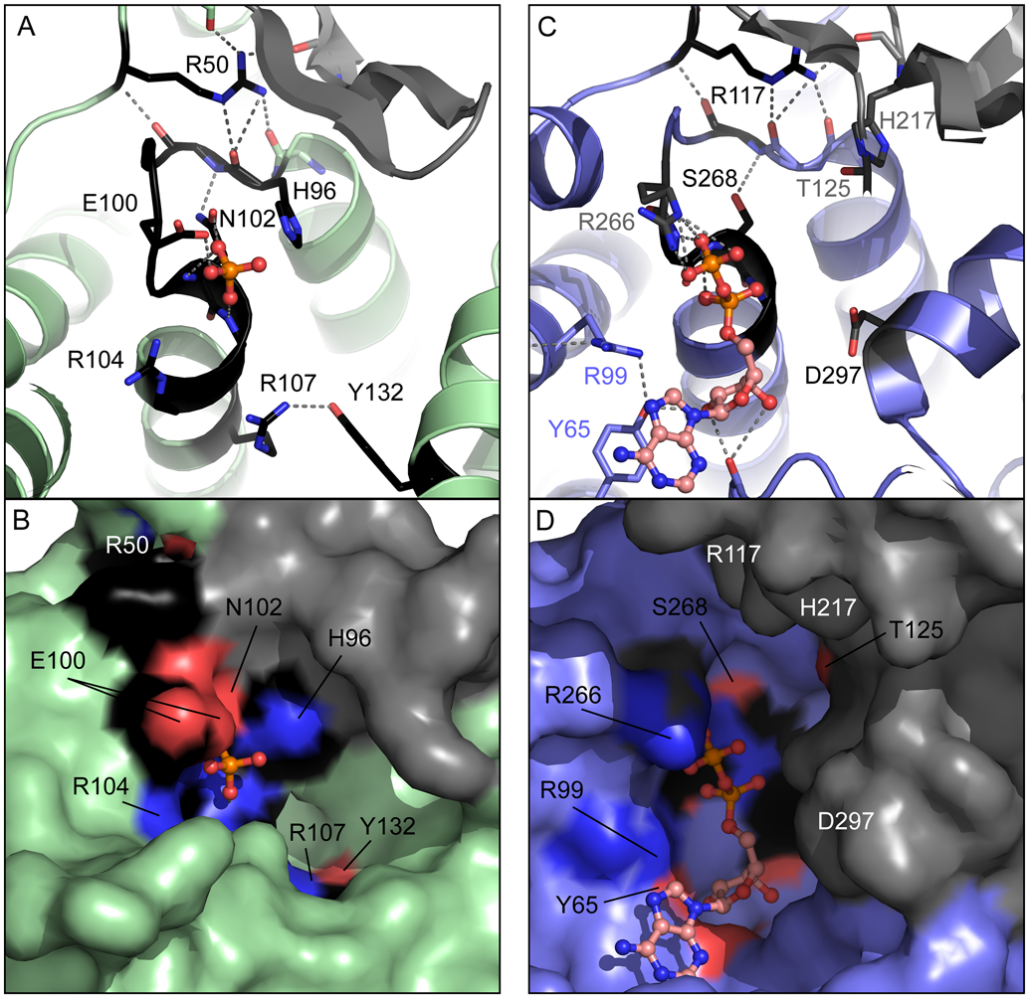
AvrB and Fic Active Site. A zoom of the active site of fic (from 2f6s) colored light green in a ribbon diagram (A) and in a surface representation (B) and the active site of AvrB (from 2nun) colored slate in a ribbon diagram (C) and in a surface representation (D). Ribbon diagrams depict conserved residues as sticks that are labeled according to the *H. pylori* fic sequence or the *P. syringae* AvrB sequence and colored according to atom: Carbon (black), Oxygen (red), Nitrogen (blue). Gray dashed lines represent hydrogen bonds between active site residues and ligands, which are depicted as ball-and-stick and colored according to atom: Phosphorous (orange), Carbon (salmon), Oxygen (red), Nitrogen (blue). An α-phosphate of the AvrB ADP binds a similar position as the fic phosphate. Similar peptide binding β-hairpins (colored gray) reside near the active site. Residues labeled in white contribute to the active site pocket with their surface rendering not visible in the depicted orientation.

Conserved fido motif residues (HPFx[D/E]GN[G/K]R) and additional conserved residues external to the motif (R50, R107, and Y132 in *H. pylori* fic) form a potential active site (Figure 4A and B). The external R50 residue organizes the fic active site, forming a number of hydrogen bonds to the backbone of the fido motif loop and to the peptide-binding β-hairpin. A phosphate residue forms hydrogen bonds to the fido motif helix backbone and the E100 and N102 side chains. A large pocket surrounds the bound phosphate, with the fido motif R104 lining a smaller cleft on one side of the bound phosphate and a Y132/R107 pair lining a larger cleft on the opposite side (Figure 4B). The smaller R104 cleft could accommodate ATP substrate phosphates, while the larger Y/R cleft could bind the sugar and nucleotide rings. The essential fido motif H96 is within 4 Å of the bound phosphate (Figure 4A) and lines a portion of the larger cleft in between the phosphate and peptide-binding β-hairpin (Figure 4B). This placement should position the fido His near the bound peptide sidechain that gets modified in the AMPylation reaction (Thr in the VopS modified Rho GTPase).

Although the AvrB sequence lacks several residues of the fido motif, a conserved Arg (R117 in *P. syringae* AvrB) that corresponds to fic R50 maintains a similar local structure in the AvrB loop (Figure 4C). The conservations of small fido motif residues that extend to this loop in AvrB help maintain the local structure. R117 also forms hydrogen bonds to the Rin4 peptide-binding β-sheet. The α-phosphate of ADP binds AvrB in a similar position as the fic phosphate, forming hydrogen bonds to the corresponding motif helix backbone. The ADP binds in a cleft formed by conserved AvrB residues and the peptide-binding β-sheet (Figure 4D). An Arg residue (R266) takes the place of the fido motif D/E and forms hydrogen bonds with the ADP β-phosphate, while the Tyr of a Tyr/Arg pair (Y65/R99) forms hydrogen bonds with one of the ribose oxygens and stacks with the nucleotide ring. The positioning of both the R266 and the Y65/R99 pair has migrated with respect to the corresponding fic residues (R104 and Y132/R107, respectively), resulting in an active site pocket that is flipped. Despite the altered orientations of the nucleotide binding pockets, the ADP α-phosphate is near the peptide-binding site. AvrB is missing the essential fido motif His. However, several conserved residues in the AvrB active site could perform a similar function: D297, T125, or H217. D297 is located near the ADP phosphate. Mutation of this residue results in a similar increase of peptide-binding affinity as mutation of the fic His [5], [19]. T125 and H217 reside in the peptide-binding β-sheet and are positioned near a Rin4 Thr bound in the active site. Mutation of either of these residues results in altered AvrB activity [18], [19].

### Activity of Fido domain-containing proteins

**Figure 5:**
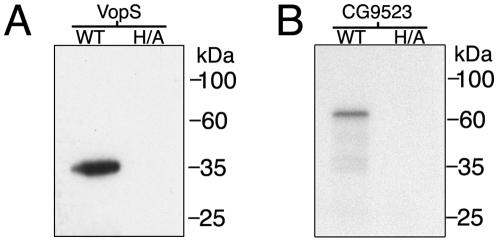
Auto-AMPylation of Fic domain-containing proteins. Recombinant VopSΔ30 (WT) or VopSΔ30-H348A (H/A) from *V. parahaemolyticus* (A) and wild-type or mutant His-tagged CG9523 from *D. melanogaster* (B) were incubated with ^32^P-α-ATP in an in vitro AMPylation assay. Samples were separated by SDS-PAGE and analyzed by autoradiography. The assay shows that wild-type, but not mutant Fic domain-containing proteins, possess auto-AMPylation activity.

A previous study showed that a secreted protein from the pathogen *V. parahaemolyticus* catalyzes the addition of adenosine 5′-monophosphate (AMP) to Rho GTPases in a process known as AMPylation [5]. This activity represents the first example of using AMP as a stable post-translational modification on a eukaryotic substrate. Recombinant VopS possesses AMPylation activity even in the absence of substrate, as it is labeled by ^32^P-α-ATP in an in vitro AMPylation assay (Figure 5A). This auto-AMPylation activity is dependent on the presence of a wild-type fic domain, as mutation of the conserved His348 residue of the fic motif to an alanine completely abrogates activity (Figure 5A). In addition, an in vitro AMPylation assay with recombinant CG9523, a fic domain-containing protein from *Drosophila melanogaster*, indicates that CG9523 possesses auto-AMPylation activity (Figure 5B). As expected, mutation of the conserved His375 to alanine leads to a loss in the auto-AMPylation activity (Figure 5B). Thus, we have extended our observations to show that both bacterial and eukaryotic fic domains mediate the novel activity of AMPylation. Future studies on this and other eukaryotic fic domain-containing proteins and the identification of AMPylated substrates will provide insight into this novel posttranslational modification mediated by the fido domain-containing family of proteins.

### Conclusion

Bacterial virulence effectors use various strategies to manipulate host cell responses, often by mimicking structures or functions of eukaryotic signaling machinery. A secreted *V. parahaemolyticus* effector VopS mediates a newly discovered posttranslational modification that stably attaches AMP to host proteins. This AMPylation activity depends on a VopS histidine residue, which belongs to a conserved sequence motif characteristic of its fic domain. We use sequence and structure based similarity detection methods to identify fic domain homologs in doc toxins and the bacterial effector AvrB. Structures of these homologs help define the active site surrounding the critical histidine and the peptide-binding mode of AMPylation targets. Finally, we demonstrate AMPylation activity of a eukaryotic fic domain, suggesting that the VopS effector has exploited a previously unknown posttranslational modification for molecular signaling.

## Materials and Methods

### Sequence similarity searches

To detect sequence homologs of fic and doc domains, we searched the non-redundant database (nrf; posted date Nov 9, 2008; 7,269,299 sequences, filtered for low complexity regions) with PSI-BLAST [20] (E-value cutoff 0.005, iterated until convergence) using query sequences corresponding to the fic domain core of the *Bacteroides thetaiotaomicron* structure 3cuc (gi|186973118, range 66 to 275) and the doc domain of the structure 3dd7 (gi| 203282431, range 1 to 126). Found homologs were grouped using linkage clustering (-lscut = 0.9), and representative sequences from each group were used as transitive queries for subsequent rounds of PSI-BLAST. To detect sequence homologs of AvrB, we searched the non-redundant database with PSI-BLAST [20] (E-value cutoff 0.02) using query sequences corresponding to the AvrB structure 1nh1 (gi| 47168369, range 1 to 320). Found homologs were grouped using linkage clustering (-lscut = 1), and representative sequences from each group were used as new queries for subsequent rounds of PSI-BLAST. The COMPASS server [22], [23] and the HHpred server [33] were used to obtain more sensitive profile-based sequence searches for various queries: AvrB (gi|149242490), duf977 (gi|169755007), duf1612 (gi|198282242, range 250–387), and duf2087 (gi|218961370); and the fold recognition meta-server 3D-jury [24] was used to identify links to a truncated AvrB query (gi|149242490, range 55–128 and range 212–321) lacking the inserted upper lobe domain. Domain organization was assessed using the conserved domain database CDD [34] with a cutoff of 0.003.

### Multiple sequence alignments and phylogenetic analysis

To visualize the relationships between identified fido sequences, all against all pair-wise sequence similarities for collected sequences (fic, doc, and AvrB) were calculated using BLAST implementation (-pval 0.1) with the CLANS application [35]. Sequences were clustered in two-dimensional space with a P-value cutoff of (1E-05) until node movement became negligible (rounds). Resulting clusters were used to constructed multiple sequence alignments using the MAFFT program (version 6, L-ins-I strategy, default parameters with no homologs [36], [37]) with manual adjustments. The multiple alignments of each group were merged into a global alignment using structure superpositions, secondary structure predictions (JPRED), hydrophobicity patterns, and paired BLAST hit alignments as guides.

### Structural similarity searches

DaliLite [38] was used to determine the closest structural neighbors in the PDB database. For each query, all pairwise comparisons were then ranked in order of descending Z-score to determine the closest structural neighbors.

### Plasmid construction and site-directed mutagenesis

CG9523 was amplified off of a plasmid containing the coding region of CG9523 (kindly provided by Helmut Kramer) using VENT polymerase. The PCR product was restriction digested, ligated into pET28a, and transformed into E. coli (DH5a, Invitrogen). Positive clones were confirmed by DNA sequence analysis. Histidine to alanine mutants of VopS and CG9523 were generated using the Stratagene QuikChangeTM site-directed mutagenesis kit, according to the manufacturer's instructions. Mutants were confirmed by DNA sequence analysis.

### Protein expression and purification

VopSΔ30 and VopSΔ30-H348A were expressed and purified as described previously [5]. Protein expression constructs pET28a-CG9523 and pET28a-CG9523-H375A were transformed into Rosetta (DE3) cells (Novagen). Single colonies were grown to an OD_600_ of 0.6–0.8 and induced with 0.4 mM IPTG for 8–12 hours at room temperature. Cells were harvested and lysed using an Emulsiflex C-5 cell homogenizer (Avastin). His-tagged proteins (CG9523 and its mutant) were purified using Ni^2+^ affinity purification (Qiagen) and were aliquoted at stored at −80°C in 20 mM Tris, pH 7.5, 5 mM NaCl, 1 mM DTT, and 10% glycerol.

### In vitro AMPylation assay

For the AMPylation assays using purified recombinant protein, 5 µM of VopSΔ30 or VopSΔ30-H348A or 2.5 µM of His-tagged CG9523 or His-tagged CG9523-H375A was incubated for 20 minutes at 30°C in 20 mM HEPES buffer containing 20 µM ATP, 2 mM MgCl_2_, and ^32^P-α-ATP (2 µCi) (Perkin Elmer). The AMPylation reaction was stopped by the addition of SDS sample buffer. Samples were boiled for 5 minutes, separated by SDS-PAGE, and visualized by autoradiography.

## Supporting Information

Figure S1 Tree illustrates extensive horizontal gene transfer in bacteria/archaea. The multiple sequence alignment of one clans group was limited to sequence ranges corresponding to the fic topology of 3cuc and purged for redundancy less than 95% identity. The resulting alignment was used to construct phylogenetic trees using the MOLPHY package [39]. JTT distances [40] were calculated using protml (-jfD options), initial neighbor-joining [41] tree topologies were built using Njdist, and maximum likelihood trees were built using local rearrangement search of initial tree topologies protlm (−R option). The tree topology reliability was assessed with estimated log-likelihood resampling (RELL) of MOLPHY.
(0.93 MB TIF)
