# Supplementary figures and images for: Fido, a Novel AMPylation Domain Common to Fic, Doc, and AvrB
Source: PLoS One. 2009 Jun 5;4(6):e5818. doi: 10.1371/journal.pone.0005818 (PMC2686095; doi:10.1371/journal.pone.0005818)

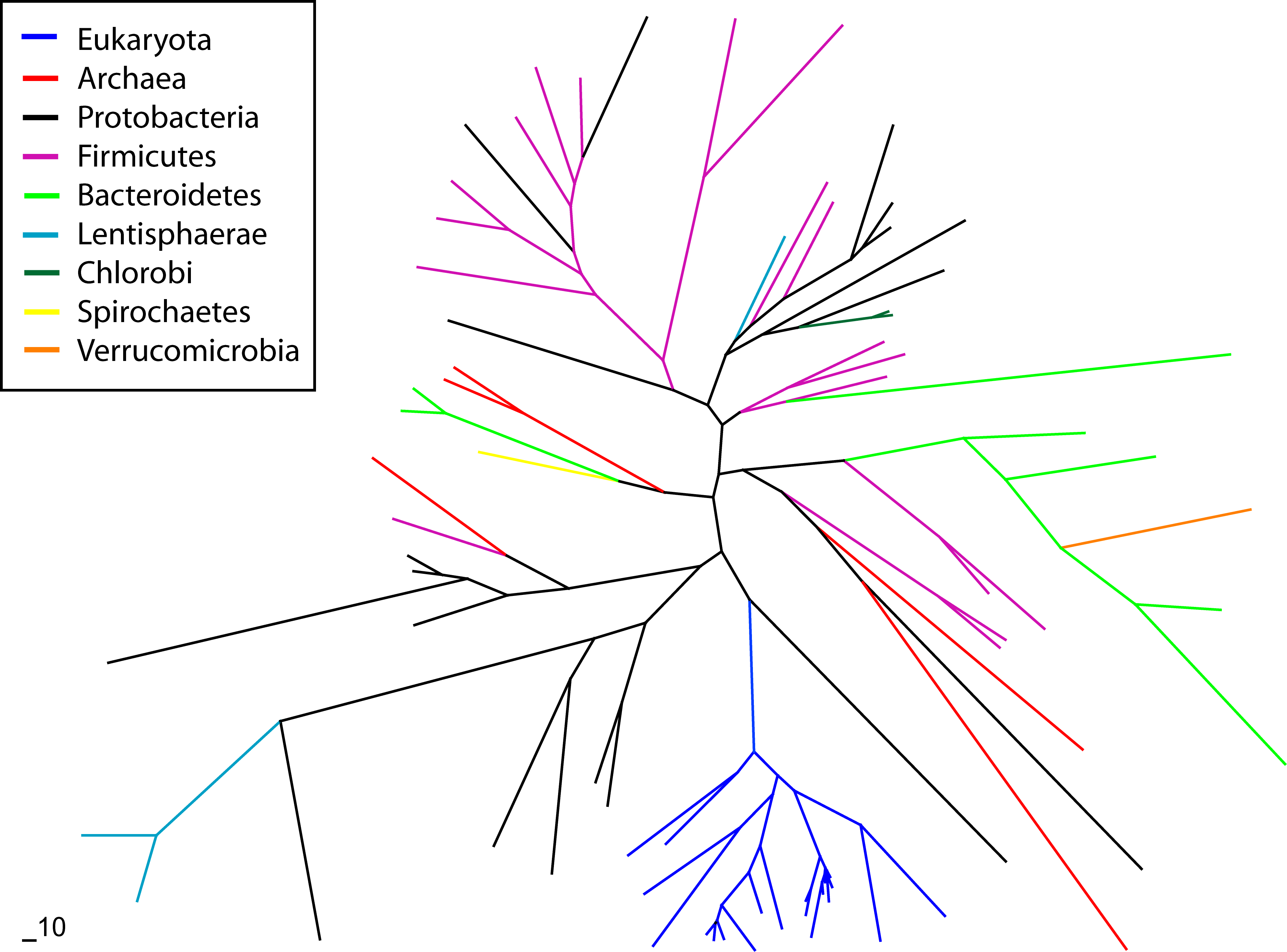

Supplement: Figure S1 — Tree illustrates extensive horizontal gene transfer in bacteria/archaea. The multiple sequence alignment of one clans group was limited to sequence ranges corresponding to the fic topology of 3cuc and purged for redundancy less than 95% identity. The resulting alignment was used to construct phylogenetic trees using the MOLPHY package [39]. JTT distances [40] were calculated using protml (-jfD options), initial neighbor-joining [41] tree topologies were built using Njdist, and maximum likelihood trees were built using local rearrangement search of initial tree topologies protlm (−R option). The tree topology reliability was assessed with estimated log-likelihood resampling (RELL) of MOLPHY. (0.93 MB TIF) [file pone.0005818.s001.tif]
